# Relationship between joint torque and muscle fascicle shortening at various joint angles and intensities in the plantar flexors

**DOI:** 10.1038/s41598-017-00485-1

**Published:** 2017-03-22

**Authors:** Atsuki Fukutani, Jun Misaki, Tadao Isaka

**Affiliations:** 1 The Research Organization of Science and Technology, Ritsumeikan University, 1-1-1 Noji-higashi, Kusatsu, Shiga 525-8577 Japan; 2 Japan Society for the Promotion of Science, Research Fellowship for Young Scientists, 5-3-1 Kojimachi, Chiyoda-ku, Tokyo 102-0083 Japan; 3 Graduate School of Sport and Health Science, Ritsumeikan University, 1-1-1 Noji-higashi, Kusatsu, Shiga 525-8577 Japan; 4 Faculty of Sport and Health Science, Ritsumeikan University, 1-1-1 Noji-higashi, Kusatsu, Shiga 525-8577 Japan

## Abstract

Because it is difficult to measure tendon length changes directly in humans, tendon length changes during dynamic movement have been evaluated indirectly from changes in muscle fascicle length and joint angle. The purpose of this study was to examine the validity of the indirect method. Twitch contractions of the ankle plantar flexors were evoked isometrically in eight subjects. Twitch contractions evoked by singlet, doublet, and triplet stimulations were conducted at dorsiflexion 20° (DF20), plantar flexion 0° (PF0), and plantar flexion 20° (PF20). Muscle fascicle length and pennation angle were recorded by ultrasonography. The magnitude of muscle fascicle shortening was significantly smaller in DF20 than in PF0 and PF20, although the magnitude of joint torque was significantly larger in DF20 than in PF0 and PF20. Theoretically, the magnitude of tendon elongation is expected to be larger in larger joint torque conditions. However, we found that the magnitude of tendon elongation evaluated from muscle fascicle shortening was larger in a lower joint torque condition (PF20). These results suggest that the magnitude of muscle fascicle shortening does not necessarily represent tendon elongation. The larger muscle fascicle shortening in PF20 may be partly caused by eliminating slack of the muscle-tendon complex.

## Introduction

During movements, a muscle generates force, which is then transmitted to a bone via a tendon. Thus, the tendon is elongated as a function of muscle force (joint torque)^1^. The changes in tendon length during movements such as running play an important role in the performance of human movements, because tendons can store and release elastic energy^2–4^.

Many studies have examined the Achilles tendon length changes during dynamic movements such as walking and jumping in humans. For example, Fukunaga *et al*.^5^ reported that during walking, the Achilles tendon was elongated and shortened instead of the muscle, which would be beneficial from the point of view of using elastic energy and optimizing muscle shortening velocity^5–8^. In these studies, the Achilles tendon length changes were estimated from changes in muscle-tendon complex length estimated from changes in knee and ankle joint angles^9, 10^, and measured muscle fascicle length changes by ultrasonography, because measuring Achilles tendon length changes directly during dynamic movements is difficult in humans. In this model, the magnitude of muscle fascicle shortening is considered to correspond with the magnitude of tendon elongation when the joint angle is constant (i.e. constant muscle-tendon complex length)^4–8^.

However, because the Achilles tendon (i.e., muscle-tendon complex) has slack^11, 12^, a muscle fascicle can be shortened by eliminating slack without storing elastic energy in the Achilles tendon. If the influence of slack were substantial, then we would have to reconsider the validity of the above method because this method to evaluate tendon length changes during dynamic movements^4–8^ does not consider the influence of slack on muscle fascicle shortening. This point should be considered to clarify the influence of elastic energy on the performance of human movements.

Thus, the aim of this study was to examine the influence of slack on the magnitude of muscle fascicle shortening, which, until now, has been considered to correspond to the magnitude of tendon elongation. To this end, we measured the magnitude of muscle fascicle shortening during twitch contractions at various joint angles and intensities. If the tendon elongates as a function of muscle force (joint torque) only at a given joint angle, then the magnitude of tendon elongation evaluated through muscle fascicle shortening should be large when the joint torque is large. On the other hand, if the influence of slack is substantial, then the magnitude of tendon elongation evaluated through muscle fascicle shortening should be large, i.e., muscle fascicle shortening is larger when the ankle joint angle is flexed (slack is prominent) even though the joint torque is smaller.

## Results

### Twitch torque

**Figure 1:**
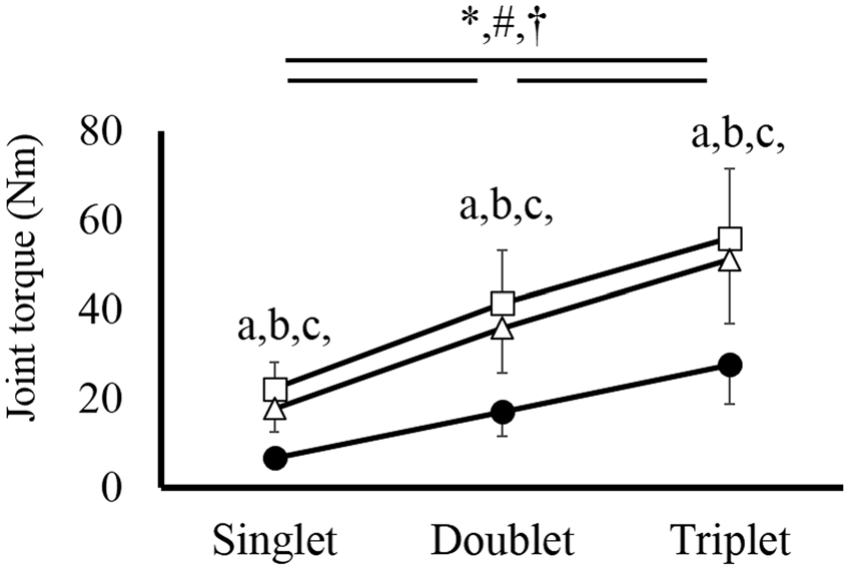
Mean twitch torque among joint angles and conditions. Mean twitch torque ± SD were plotted against twitch contraction at a given joint angle: DF20, white squares; PF0, white triangles; PF20, black circles. (a) Significant difference between DF20 and PF0 (*P* < 0.05). (b) Significant difference between DF20 and PF20 (*P* < 0.05). (c) Significant difference between PF0 and PF20 (*P* < 0.05). *Significant difference among conditions in DF20 (*P* < 0.05); ^#^Significant difference among conditions in PF0 (*P* < 0.05); ^†^Significant difference among conditions in PF20 (*P* < 0.05).

Significant interaction (joint angle × condition) was found in the twitch torque (*F* value = 56.067, *P* < 0.001, *η* ^*2*^ = 0.889). Subsequent analyses revealed that the magnitude of the twitch torque was significantly larger in DF20 than in PF0 and in PF20 at all conditions (singlet, doublet, triplet) (*P* < 0.001–*P* = 0.029). In addition, the magnitude of the twitch torque was significantly larger in triplet than in doublet and singlet, and smaller in singlet than in doublet and triplet conditions in all joint angles (*P* < 0.001) (Fig. 1).

### Fascicle shortening

**Figure 2:**
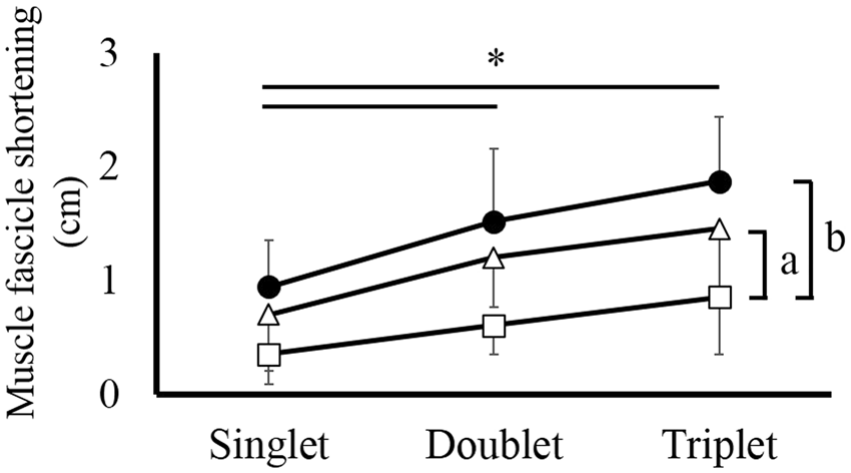
Fascicle shortening among joint angles and conditions. Mean fascicle length ± SD measured at each twitch contraction condition: DF20, white squares; PF0, white triangles; PF20, black circles. (a) Significant difference between DF20 and PF0 (*P* < 0.05). (b) Significant difference between DF20 and PF20 (*P* < 0.05). *Significant difference among conditions (*P* < 0.05).

No significant interaction (joint angle × condition) was found in the muscle fascicle shortening, which is an index of tendon elongation (*F* value = 0.786, *P* = 0.50, *η* ^*2*^ = 0.101), but a significant main effect was found in the joint angle (DF20, PF0, PF20) and condition (singlet, doublet, triplet). Subsequent analyses revealed that the magnitude of muscle fascicle shortening was smaller in DF20 than in PF0 (*P* = 0.003) and in PF20 (*P* < 0.001), and smaller in the singlet condition than in PF0 (*P* = 0.001) and in PF20 (*P* = 0.009). No significant difference was found between PF0 and PF20 (*P* = 0.225) nor between the doublet and triplet conditions (*P* > 0.999) (Fig. 2).

## Discussion

The aim of this study was to examine whether slack had any influence on muscle fascicle shortening, which is considered to correspond to tendon elongation. To accomplish this aim, the magnitude of muscle fascicle shortening was compared among DF20, PF0, and PF20. Our results demonstrated that the magnitude of muscle fascicle shortening was significantly smaller in DF20 than in PF0 and PF20 although joint torque was significantly larger in DF20 than in PF0 and PF20. Theoretically, the magnitude of tendon elongation is expected to be larger in larger joint torque conditions. However, we found that the magnitude of tendon elongation evaluated from muscle fascicle shortening was larger in lower joint torque conditions (PF20). These results suggest that the magnitude of muscle fascicle shortening does not necessarily represent tendon elongation.

**Figure 3:**
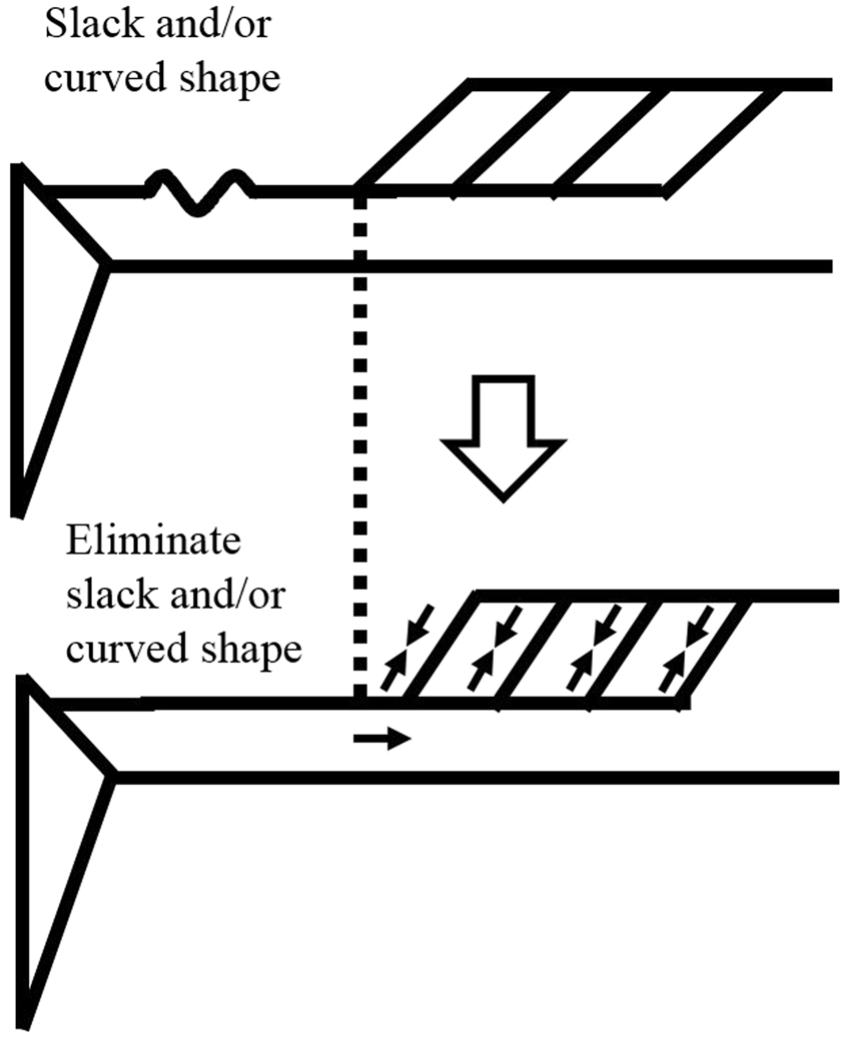
Schematic representation of the interaction between slack and/or curved shape and muscle fascicle shortening at a given ankle joint angle.

Because of the force-length relationship of the muscle^13, 14^ and the limited operating range of the medial gastrocnemius (i.e., ascending limb only)^15, 16^, muscle force (joint torque) should be larger in DF20 than in PF0 or PF20. Thus, the magnitude of tendon elongation should be larger in DF20 than in PF20 according to the load-elongation relationship of the tendon^1^. However, the magnitude of tendon elongation evaluated through muscle fascicle shortening was smaller in DF20 than in PF20 (Fig. 2). This would be caused by the influence of slack of the muscle-tendon complex^11, 12^. If slack exists in a series elastic elements (not only the tendon but also the aponeurosis and muscle), the muscle fascicle can shorten during muscle contractions without significant tendon elongation (Fig. 3) at a given joint angle. This means that elastic energy cannot be stored in the tendon although substantial muscle fascicle shortening is observed. In addition to slack, the curved shape of the Achilles tendon in the plantar flexion region^17, 18^ would also function similar to slack, because a muscle force applied to the Achilles tendon straightens the curved shape, leading to shortening of the muscle fascicle even without tendon elongation.

**Figure 4:**
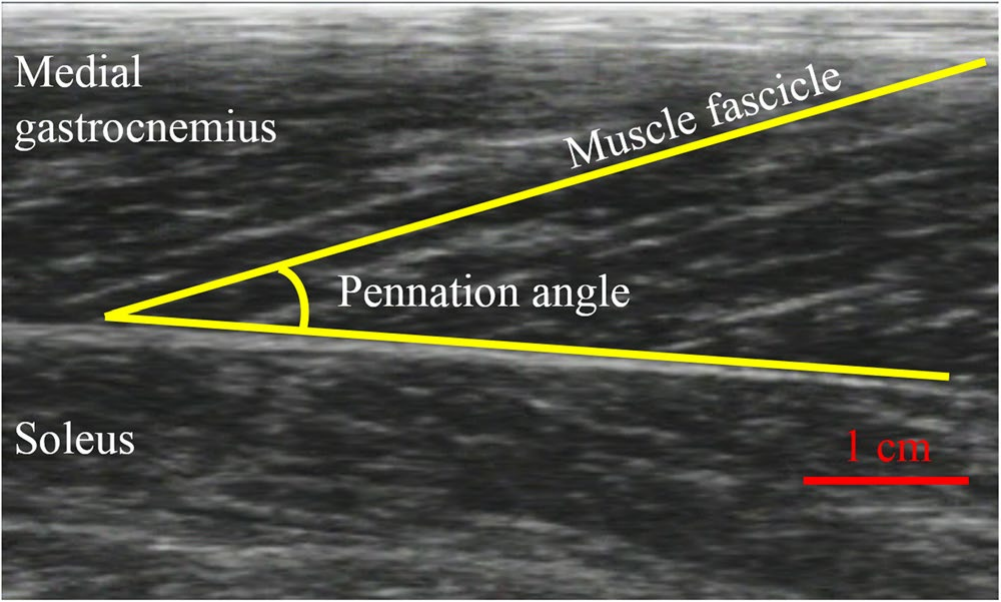
Illustration of how muscle fascicle length and pennation angle are measured.

As is the case with previous studies^4–8^, our recent studies confirmed that muscle fascicle behavior does not necessarily correspond with joint angle changes^19, 20^. Previously, these phenomena were explained by tendon elongation, and considered to be directly linked with storing and releasing elastic energy. However, this study suggests that care must be taken to estimate tendon length changes from muscle fascicle changes because there is a possibility that eliminating slack of the muscle-tendon complex results in muscle fascicle shortening (Fig. 4). This influence should be prominent when the ankle joint operates under the plantar flexion region (i.e., shortened position) because the influences of slack^11^ and curved shape^18^ should be prominent. Thus, from the point of view of storing and releasing elastic energy, the influences of eliminating slack and/or curved shape on the magnitude of muscle fascicle shortening should be considered. On the other hand, from the point of view of optimizing muscle fascicle behavior, which is also called muscle-tendon interaction^21–23^, the influences of eliminating slack and/or curved shape would not be a concern. This is because optimizing muscle fascicle behavior occurs not only by tendon elongation (muscle-tendon interaction) but also eliminating slack and/or curved shape (muscle-slack and/or curved shape interaction). Thus, eliminating slack would have substantial influence on the force-generating capability of muscle from the view of modulating muscle fascicle length changes.

It is difficult to clarify the extent of influence of slack and/or curved shape and tendon elongation on muscle fascicle shortening, but is worth mentioning. In this study, the magnitude of joint torque was similar between the triplet condition at PF20 (27.7 ± 8.8 Nm) and the singlet condition at DF20 (22.2 ± 6.1 Nm), although the magnitude of fascicle shortening was clearly different between the former condition (1.9 ± 0.6 cm) and the latter condition (0.4 ± 0.3 cm). Thus, if we assume that at DF20, the influences of slack and/or curved shape were zero and that tendon elongation (0.4 cm) was caused by muscle force only (i.e., 22.2 Nm elongated tendon by 0.4 cm), an elongation of 0.4 cm out of a total of 1.9 cm (20%) should be caused by muscle force and an elongation of 1.5 cm out of a total of 1.9 cm (80%) should be caused by eliminating slack and/or curved shape at PF20. Thus, the influence of slack should be substantial at least in the plantar flexion region.

There are some limitations in this study. First, the contraction intensities adopted in this study was limited to relatively low intensities, i.e., singlet, doublet, and triplet. In the case of higher intensity contractions, larger tendon elongation should be expected. However, the influence of slack and curved shape on the muscle fascicle shortening should exist irrespective of contraction intensities because these influences are caused by the geometric characteristics of plantar flexors. Thus, we believe that our conclusion would be applicable even in the case of higher intensity contractions. On the other hand, it is not sure whether our conclusion is applicable to other muscles/joints. As discussed, the reason why the magnitude of muscle fascicle shortening was independent of joint torque should be slack and/or curved shape of muscle tendon unit. Although the influence of slack would exist in other muscle tendon units such as knee extensors and elbow extensors, the influence of curved shape is limited only in the plantar flexors because curved shape is a specific characteristics of plantar flexors. Therefore, similar examination adopting other muscles should be conducted to extend our concept to other muscles.

## Conclusion

Tendon elongation evaluated through muscle fascicle shortening was measured in DF20, PF0, and PF20 at singlet, doublet, and triplet conditions. As a result, in PF20, the magnitude of muscle fascicle shortening was larger although joint torque was smaller than in PF0 and in DF20. These results indicate that the magnitude of tendon elongation evaluated through muscle fascicle shortening should be affected by slack in the muscle-tendon complex. Thus, from the point of view of using elastic energy, care should be taken when interpreting the meaning of tendon elongation evaluated indirectly through muscle fascicle shortening.

## Methods

### Subjects

Eight men (age, 23.6 ± 2.2 years; height, 1.69 ± 0.02 m; body mass, 65.3 ± 4.7 kg) participated in this study. The purpose and risks of this study were explained to each participant, and written informed consent was obtained from all participants. The Ethics Committee on Human Research of Ritsumeikan University approved this study (IRB-2014-026). This study was conducted according to the principles expressed in the Declaration of Helsinki.

### Experimental procedures and torque measurement

The right leg was adopted as a testing target. Subjects were fixed to the dynamometer (Biodex; SAKAImed, Tokyo, Japan) with the hip and knee joints being 0° (the anatomical position was defined as 0° for both hip and knee joints). The ankle joint was set at dorsiflexion 20° (DF20), plantar flexion 0° (PF0), and plantar flexion 20° (PF20). Twitch contractions were evoked at these three joint angles. The stimulating electrodes were placed just above the patella (4 × 5 cm) and popliteal fossa (1 × 1 cm) to stimulate the tibial nerve. The locations of the electrodes were adjusted to obtain large twitch responses with minimum stimulation voltage. The intensity of twitch contraction was supramaximal (i.e. 20% higher than the intensity in which maximal twitch torque was obtained), and the pulse duration of electrical stimulation was 0.5 ms. Three types of twitch contractions were adopted in each joint angle: singlet, doublet, and triplet. The stimulation frequency was set at 100 Hz. The twitch contractions were evoked in the following order: singlet, doublet, and triplet with a 30 s interval between contractions. Subsequently, the next joint angle condition was tested with an interval of more than 120 s. The sequence was randomized. Peak torque attained during twitch contractions was adopted as the twitch torque. Passive torque was subtracted to calculate active torque. This value was used in the following analyses as twitch torque.

### Ultrasonographic measurement

Ultrasonography (SSD-3500; Aloka, Tokyo, Japan) with a linear array probe (UST-5710; Aloka, Tokyo, Japan) was used to obtain images of the muscle belly of the medial gastrocnemius (Fig. 4) with a sampling frequency of 30 Hz. Muscle fascicle length and pennation angle during each twitch contraction were obtained. Muscle fascicle length was defined as the distance between the intersection composed of the superficial aponeurosis and muscle fascicle, and the intersection composed of the deep aponeurosis and muscle fascicle. Pennation angle was defined as the angle between the muscle fascicle and deep aponeurosis. The magnitude of muscle fascicle shortening during twitch contractions (i.e., muscle fascicle length at rest minus muscle fascicle length at the moment when peak twitch torque occurred) was calculated. This value was corrected by considering the pennation angle obtained at the moment when peak twitch torque occurred (i.e., muscle fascicle length × cos θ). This muscle fascicle shortening is considered to correspond to the tendon elongation when the joint angle (i.e., muscle-tendon complex length) is constant^5–8^. In our previous study, coefficient of variations of fascicle length and pennation angle were confirmed to be 1.1% and 1.6%, respectively, while the intraclass correlation of those were confirmed to be 0.993 and 0.989, respectively^24^.

### Statistical analysis

Descriptive data are presented as means ± standard deviations. Two-way analysis of variance (ANOVA) with repeated measure was conducted to confirm an interaction (joint angle × condition) and a main effect. If significant interaction was found, one-way ANOVA with repeated measure and following post hoc test (Bonferroni correction) were conducted. The level of statistical significance was set at *P* < 0.05. Statistical analysis was conducted using IBM SPSS Statistics (Version 20.0, IBM, Tokyo, Japan).
